# CdTe X/γ-ray Detectors with Different Contact Materials

**DOI:** 10.3390/s21103518

**Published:** 2021-05-18

**Authors:** Volodymyr Gnatyuk, Olena Maslyanchuk, Mykhailo Solovan, Viktor Brus, Toru Aoki

**Affiliations:** 1 V.E. Lashkaryov Institute of Semiconductor Physics of the National Academy of Sciences of Ukraine, Prospekt Nauky 41, 03028 Kyiv, Ukraine; 2 Institute of Applied Physics and Computer Sciences, Yuriy Fedkovych Chernivtsi National University, Kotsyubynskyi Str. 2, 58012 Chernivtsi, Ukraine; m.solovan@chnu.edu.ua; 3 Department of Physics, School of Sciences and Humanities, Nazarbayev University, Kabanbay Batyr Ave 53, Nur-Sultan 010000, Kazakhstan; viktor.brus@nu.edu.kz; 4 Research Institute of Electronics, Shizuoka University, 3-5-1 Johoku, Naka-ku, Hamamatsu 432 8011, Japan; aoki.toru@shizuoka.ac.jp

**Keywords:** CdTe detectors, X-ray and γ-ray spectroscopy, Schottky contact, *p-n* junction, charge transport mechanism

## Abstract

Different contact materials and optimization of techniques of their depositions expand the possibilities to obtain high performance room temperature CdTe-based X/γ-ray detectors. The heterostructures with ohmic (MoO*_x_*) and Schottky (MoO*_x_*, TiO*_x_*, TiN, and In) contacts, created by DC reactive magnetron sputtering and vacuum thermal evaporation, as well as In/CdTe/Au diodes with a *p-n* junction, formed by laser-induced doping, have been developed and investigated. Depending on the surface pre-treatment of semi-insulating *p*-CdTe crystals, the deposition of a MoO*_x_* film formed either ohmic or Schottky contacts. Based on the calculations and *I-V* characteristics of the Mo-MoO*_x_*/*p*-CdTe/MoO*_x_*-Mo, In/*p*-CdTe/MoO*_x_*-Mo, Ti-TiO*_x_*/*p*-CdTe/MoO*_x_*-Mo, and Ti-TiN/*p*-CdTe/MoO*_x_*-Mo Schottky-diode detectors, the current transport processes were described in the models of the carrier generation–recombination within the space-charge region (SCR) at low bias, and space-charge limited current incorporating the Poole–Frenkel effect at higher voltages, respectively. The energies of generation–recombination centers, density of trapping centers, and effective carrier lifetimes were determined. Nanosecond laser irradiation of the In electrode, pre-deposited on the *p*-CdTe crystals, resulted in extending the voltage range, corresponding to the carrier generation–recombination in the SCR in the *I-V* characteristics of the In/CdTe/Au diodes. Such In/CdTe/Au *p-n* junction diode detectors demonstrated high energy resolutions (7%@59.5 keV, 4%@122 keV, and 1.6%@662 keV).

## 1. Introduction

One of the key properties of semiconductors, known as the photo-effect, makes these materials the best candidates for photo-sensors for a wide spectral range (from γ-rays to terahertz radiation). Indeed, thanks to the great ability to directly convert photons to electric charge carriers, i.e., an electrical signal, semiconductor detectors have significant advantages compared with other ionizing radiation sensors such as scintillators, gas-based detectors, etc. [1]. The favorable features of semiconductor nuclear radiation detectors are: higher energy and spatial resolution, sufficient detection efficiency, satisfactory rate and timing characteristics, enhanced imaging capabilities, and promising ability to be fabricated as compact sensor modules for portable ionizing radiation detecting instruments operating at room temperature [1,2].

Among many suitable semiconductors, used for X/γ-ray (in other words, high-energy, nuclear, or ionizing radiation) detectors, cadmium telluride (CdTe) is the most studied, employed, and attractive compound because of its favorable physical properties [2,3,4,5,6]. Indeed, an optimal set of electrical and electronic characteristics of high-resistivity CdTe makes this material basic and still promising for compact uncooled X/γ-ray detectors covering a wide energy range from a few keV to tens MeV, which are widely used in science, industry, security, ecology, medicine, space astronomy and many other application fields [3,4,5,6,7,8]. Among the excellent features of this semiconductor, there are key advantages that should be highlighted: large atomic numbers of the compound components (*Z*_Cd_ = 48 and *Z*_Te_ = 52) provide high absorption efficiency for X/γ-photons, stopping power and, therefore, high radiation attenuation coefficient; wide bandgap energy (*E*_g_ ~ 1.5 eV) and therefore, fairly high electrical resistivity (ρ > 10^9^ Ω·cm) along with suitable charge transport properties allow CdTe-based detectors to operate without liquid nitrogen or Peltier cooling [3,4,5,6,7,8,9,10]. As a result, uncooled CdTe-based detectors make it possible to achieve high radiation detection efficiency, energy resolution, and fast timing response to be efficiently employed in portable detecting devices [5,6,7,8,9,10].

High-resistivity CdTe single crystals have been successfully used in the fabrication of room temperature X/γ-ray detectors in two principally different ways (basic technologies): (i) creation of two ohmic electrical contacts; (ii) formation of diode structures either with a Schottky contact or *p-n* junction [3,4,5,6,7,8,9,10]. CdTe detectors with two ohmic contacts operate at low bias voltage and have good time stability of functional parameters (detection efficiency and energy resolution). However, ohmic contact detectors biased with low (tens of volts) voltages suffer from an incomplete collection of radiation-generated carriers and, hence, a significant amount of charge losses because holes can be trapped before reaching the cathode. Therefore, the energy resolution of ohmic detectors is not high enough, but higher bias voltage (hundreds of volts) cannot be applied because of an increase in leakage (dark) current [5,6,7,8,9,10].

Dark current can be significantly decreased by replacing one ohmic contact with a Schottky-barrier contact or creating a *p-n* junction. In so doing, it is possible to increase applied voltage (up to thousands of volts) because diode-type detectors operate at reverse bias. Increasing the electric field extends the depletion region, and thus improves the charge collection in diode detectors. This ensures higher energy resolution and detection efficiency [5,6,7,8,9,10]. Such possibilities of creating CdTe-based barrier structures for X/γ-ray detectors have been discovered and used since the 1960s [3,11]. However, CdTe diode-type detectors suffer from the charge polarization effect which is one of the important problems that degrades detector parameters with operation time and limits their practical application [3,4,5,6,7,8,12,13,14]. Therefore, both the basic techniques have been developed and presently employed to produce both ohmic and diode-type detectors, respectively [5,6,7,8,9,10,12,13,14].

At the end of the 1990s, Acrorad Co. Ltd. (Okinawa, Japan) succeeded in growing high-quality bulk semi-insulating CdTe semiconductor ingots and employed indium as a low work-function metal to form a Schottky contact with a high barrier for holes at the CdTe crystal surface. As a result, the In/CdTe/Pt Schottky-diode X/γ-ray detectors with high energy resolution were obtained [7,8,9,10]. Since that time, CdTe crystals and Schottky detectors produced by Acrorad have been widely used by many investigators and experts in the world. Aluminum has been also employed to form a Schottky contact because both metals (In and Al) belong to Group III of the periodic table and have similar characteristics to be suitable for creation of a high Schottky barrier [12,13,14]. Furthermore, Al as metal with the high melting temperature is more suitable in cases of solder bumping and wire bonding fabrication as well as other procedures requiring heating of diodes.

During the last two decades, we have widely used semi-insulating CdTe semiconductors, produced by Acrorad for material characterization [15,16,17] and elaboration of both Schottky diode-type [18,19,20,21,22,23,24,25,26,27] and M-*p-n* structured [27,28,29,30,31,32,33,34] X/γ-ray detectors. Using different metals (Ni, Cr, In, Al, and Au) for electrodes to form appropriate Schottky and ohmic electrical contacts or employing laser-induced doping of a thin CdTe layer with In, we have obtained high performance diode-type X/γ-ray detectors with excellent room-temperature energy resolution. The Ni/CdTe/Ni Schottky-diode detectors, fabricated using Ar plasma treatment, and the In/CdTe/Au *p-n* junction-diode detectors formed by the laser-assisted technique, demonstrated FWHM ≈ 0.5–1%@662 keV [18,19,20,27] and FWHM ≈ 0.7–1.4%@662 keV [27,34], respectively.

We have recently studied other electrode materials (graphene, metal oxides and nitrides) different from metals used before and developed the techniques to employ them for the formation of Schottky and ohmic electrical contacts to Acrorad’s semi-insulating CdTe crystals [21,22,23,24,25,26]. We have interested to develop this trend, in particular using molybdenum oxide as a promising material for ohmic contact formation [35,36,37,38,39,40]. In addition, titanium oxide, titanium nitride, and indium have been employed to create Schottky contacts. We also fabricated CdTe-based M-*p-n* structured diodes, developing and applying the laser solid-phase doping technique [33,34]. The aim of this paper is to review our experience in the creation and study of CdTe-based structures for X/γ-ray detectors using the same semiconductor material, i.e., detector-grade *p*-like CdTe crystals produced by Acrorad [8,9,10].

## 2. Semiconductor Samples

To meet the requirements for materials suitable for fabrication of room temperature X/γ-ray detectors, we have used commercial detector-grade *p*-like CdTe semiconductor crystals, produced by Acrorad Co., Ltd. [8,9,10]. Semi-insulating CdTe semiconductor has been obtained by the Traveling Heater Method (THM), which is known as an advanced and efficient technique to grow high-quality single crystals (with chemical purity, higher uniformity, structural perfection, reduced number of extended defects as well as relatively low concentration of native point defects and accidental impurities, etc.) [4,5,6,7,8,9,10].

High-resistivity Cl-compensated CdTe crystals showed weak *p*-type conduction with room temperature resistivity ρ = (2–4) × 10^9^ Ω·cm that was close or even higher than the intrinsic value (ρ_i_ ≈ 4 × 10^9^ Ω·cm) [16,17,18]. The estimated electron and hole densities were *n* ≈ 4.2 × 10^4^ cm^−3^ and *p* ≈ 2.6 × 10^7^ cm^−3^ [9,10]. It was shown that the employed CdTe could be considered as an almost intrinsic semiconductor with the bandgap energy *E*_g_ = 1.47 eV (at *T* = 300 K), effective masses of electrons *m*_n_ = 0.11 *m*_0_ and holes *m*_p_ = 0.53 *m*_0_, where *m*_0_ is the free electron mass [15,16].

The grown CdTe ingots were sliced into (111) oriented single-crystal wafers. The (111) crystal surfaces of CdTe semiconductor exhibit crystallographic polarity. Usually, the close packed plane terminated by Cd atoms is called the *A*-face, whereas that terminated by Te atoms is called the *B*-face [3,4,5,6]. Conventionally, many properties of polar surfaces (CdTe(111)*A* and CdTe(111)*B*) differ considerably. Parallelepiped-like (111) oriented CdTe samples with a surface area of 5 × 5 mm^2^ and thickness of 0.5 mm or 0.75 mm, preliminary polished by the manufacturer, were used in our experiments to fabricate CdTe-based heterostructures, Schottky diodes and M-*p-n* structures with different electrical contacts which then were tested as X/γ-ray detectors. Prior to employing the technological procedures of modification of the surface states, formation of electrical contacts, and electrode deposition, CdTe single-crystal wafers were subjected to preliminary surface processing for removing surface contaminations and cleaning. The conditions and features of surface treatments for each case have been described in the corresponding sections below.

## 3. Capabilities of CdTe-Based X/γ-ray Detectors with MoO*_x_* Ohmic Contacts

### 3.1. Fabrication of CdTe-Based Detectors with MoO_x_ Ohmic Contacts

#### 3.1.1. Molybdenum Oxide as a Prospective Material for Ohmic Contact Formation

Molybdenum oxide (MoO*_x_*) has been widely used as an interlayer for the fabrication of high performance electrical contacts to low resistance *p*-CdTe semiconductor [35,36,37], in particular as a material with large work function [38,39,40]. Moreover, MoO*_x_* is favorably distinguished by its high transparency for visible radiation and relatively low specific electrical resistivity. Due to these properties and high work function (5.2–6 eV) [37], MoO*_x_* has been considered and studied as a promising candidate to form efficient electrical contacts to semi-insulating *p*-CdTe semiconductor crystals [21,22,23,24,25].

Prior to the formation of electrical contacts and electrode deposition, the investigated CdTe crystals were subjected to preliminary chemical polishing etching in a solution of K_2_Cr_2_O_7_ + HNO_3_ + H_2_O for 20–30 s. For fabrication of Mo-MoO*_x_*/*p*-CdTe/MoO*_x_*-Mo X/γ-ray detectors with ohmic contacts, MoO*_x_* films were deposited on the pre-heated up to ~100 °C surface of the CdTe substrates in a universal vacuum system Leybold-Heraeus L560 by DC reactive magnetron sputtering of a pure molybdenum target in an argon-oxygen mixture atmosphere for ~5 min. Prior to the electrode deposition process, the vacuum chamber was evacuated to a residual pressure of 5 mPa and the *p*-CdTe substrate surface was subjected to relatively long-term bombardment by Ar ions (ion beam intensity was ~15 mA/cm^2^ and processing time was ~10 min) to create a *p*^+^-layer, which increased the ohmic contact quality. The partial pressures of argon and oxygen in the vacuum chamber during the MoO*_x_* film deposition were 240 and 24 mPa, respectively. The magnetron power was ~120 W. After the deposition of a MoO*_x_* film, the oxygen supply was closed and deposition of a pure molybdenum film was carried out for ~1 min. Thanks to the optimal relationship of the work functions of *p*-CdTe and MoO*_x_*, and the created *p*^+^-layer with an increased concentration of uncompensated acceptors, the Mo-MoO*_x_*/*p*-CdTe/MoO*_x_*-Mo ohmic structures with a minimum potential barrier at the contacts were formed.

**Figure 1:**
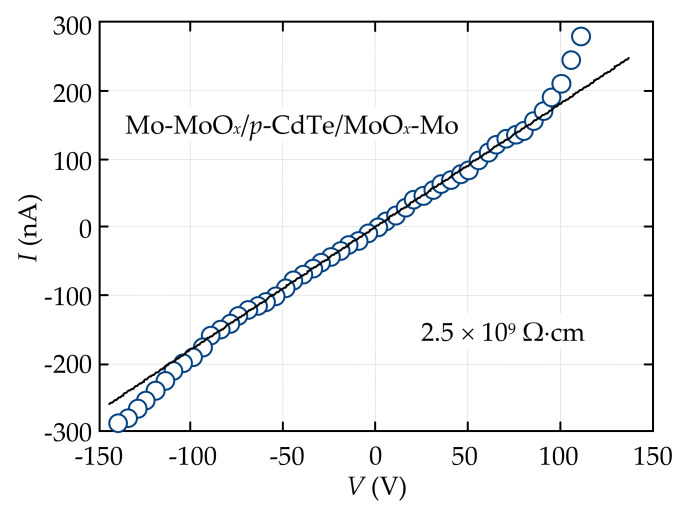
*I-V* characteristics of the Mo-MoO*_x_*/*p*-CdTe/MoO*_x_*-Mo detector with two ohmic contacts at both polarities of applied voltage (circles). The solid line demonstrates a linear voltage dependence of the current.

The fabricated Mo-MoO*_x_*/*p*-CdTe/MoO*_x_*-Mo ohmic detectors were characterized by electrical and spectroscopic measurements at room temperature. As seen from Figure 1, the *I-V* characteristic of the Mo-MoO*_x_*/*p*-CdTe/MoO*_x_*-Mo sample, measured with a delay time of 1.5 s, is symmetric and follows Ohm’s law in the entire range of applied bias voltages. Such a feature confirms the formation of a high-quality ohmic contact at the MoO*_x_*/*p*-CdTe interface. The slope of the voltage dependence of the current corresponds to a specific resistivity of 2.5 × 10^9^ Ω⋅cm, which is a typical value for detector-grade high-resistivity CdTe material grown by Acrorad Co. Ltd. [8,9,10,16]. It should be noted that a sharper increase in the current is observed at *|V*| > ±100 V (Figure 1). This feature of the *I-V* characteristic, which exhibits a non-ohmic behavior of the MoO*_x_*/*p*-CdTe contacts, is discussed in detail in Section 3.2.

#### 3.1.2. Schottky Contact Formation

**Figure 2:**
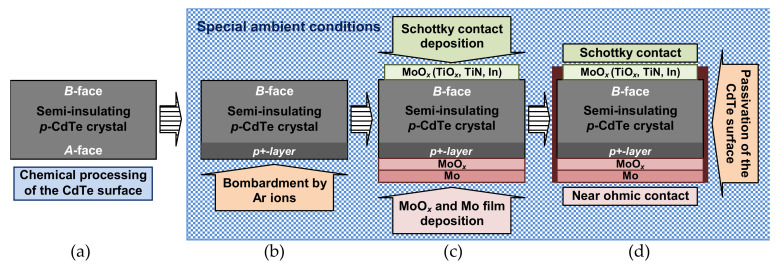
Schematic illustration of the fabrication procedures of MoO*_x_*(TiO*_x_*, TiN, In)/*p*-CdTe/MoO*_x_* diode detectors: (**a**) chemical surface processing of the crystal; (**b**) bombardment by Ar ions to create a *p*^+^-layer on the CdTe(111)*A* surface; (**c**) Schottky (ohmic) contact formation; (**d**) passivation of the crystal surfaces.

Schottky and ohmic contacts were formed on the CdTe(111)*B* side (Te-terminated) and CdTe(111)*A* side (Cd-terminated) of the crystals, respectively (Figure 2). The different conditions under Ar ion etching of the CdTe crystal surfaces were used before the electrode deposition and creation of Schottky and ohmic contacts. In particular, prior to the deposition of MoO*_x_*, TiO*_x_*, and TiN films, the *B*-face of the CdTe substrates was bombarded with Ar ions with the intensity of the ion beam about 5 mA/cm^2^ (Figure 2b). The accelerating voltage was ~1000 V and the etching time was about 300 s. The distinctions between the properties of the junctions (electrical contacts), formed on the opposite sides of the CdTe(111) single-crystal wafers, were attributed to the modification of the system of electronic states and the Fermi level pinning at the *A*-face (Cd-terminated) and *B*–face (Te-terminated).

The MoO*_x_* and TiO*_x_* films were deposited by DC reactive magnetron sputtering of a pure molybdenum or titanium target at the CdTe substrate temperature ~100 °C in an argon-oxygen mixture atmosphere (partial pressures of argon and oxygen in the vacuum chamber were 240 mPa and 24 mPa, respectively) with the processing time of ~60 s (Figure 2c). Whereas a TiN thin film was deposited in an argon–nitrogen mixture atmosphere (partial pressures of argon and nitrogen were 350 mPa and 700 mPa, respectively) for ~15 min. Since the MoO*_x_*, TiO*_x_*, and TiN films, deposited by DC reactive magnetron sputtering, showed high electrical conductivity, we considered the MoO*_x_*/*p*-CdTe, TiO*_x_*/*p*-CdTe, and TiN/*p*-CdTe heterostructures as Schottky-type contacts. To form an In/*p*-CdTe Schottky contact, an In film was deposited by vacuum thermal evaporation of pure indium from a tungsten crucible. All the electrodes were circular with a diameter of 4 mm and were centered on the Te- and Cd-terminated surfaces of the CdTe(111) crystals (5 × 5 mm^2^ squares), forming Schottky or ohmic contacts, respectively.

Afterward, the edges of the CdTe-based structures were coated with an aluminum oxide (Al_2_O_3_) layer in order to passivate surface defects on the CdTe crystals, minimize undesirable surface and lateral leakage currents and protect the semiconductor surface from degradation (Figure 2d). An Al_2_O_3_ thin film was deposited onto the formed CdTe heterostructures by the DC reactive magnetron sputtering technique in an argon–oxygen mixture atmosphere. The partial pressures of both argon and oxygen in the vacuum chamber during the deposition process were 400 mPa. The magnetron power was ~150 W. The deposition process lasted for 10 min at the CdTe substrate temperature of ~100 °C.

### 3.2. Electrical Characteristics of CdTe-Based Detectors with MoO_x_ Ohmic Contacts

#### 3.2.1. *I-V* Characteristics of the Heterostructures with MoO*_x_* Ohmic Contacts

**Figure 3:**
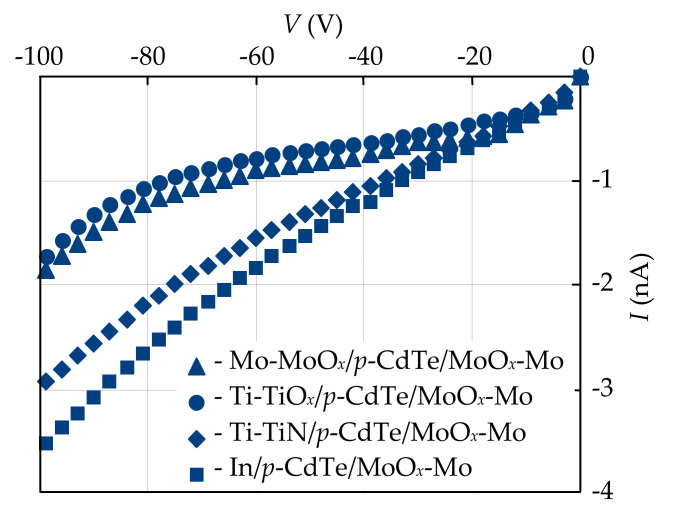
Reverse *I-V* characteristics of the CdTe-based Schottky-diode detectors with one MoO*_x_* contact and the second one made from different materials: MoO*_x_* (triangles), TiO*_x_* (circles), TiN (diamonds), and In (squares).

The fabricated CdTe-based detectors with different Schottky contacts and MoO*_x_* ohmic contacts were characterized by electrical measurements in the dark at different temperatures by a standard method with the use of a precise femto/picoammeter Keysight B2985A with a built-in voltage source (±1000 V) and Agilent 34410A as a voltmeter. The *I-V* characteristics of the fabricated Mo-MoO*_x_*/*p*-CdTe/MoO*_x_*-Mo, Ti-TiO*_x_*/*p*-CdTe/MoO*_x_*-Mo, Ti-TiN/*p*-CdTe/MoO*_x_*-Mo, and In/*p*-CdTe/MoO*_x_*-Mo heterostructures showed rectification properties. In terms of the practical application of the developed CdTe-based structures for detection of X/γ-ray radiation, it is important to analyze the reverse branches of the *I-V* characteristics when the Schottky contact is biased positively with respect to the MoO*_x_*-Mo ohmic contact because of diode-type detectors operate under reverse bias [4,5,6,7,8]. It should be noted that the reverse currents in the heterostructures under investigation are equal to several nanoamperes at bias voltages of ~50–100 V at room temperature (Figure 3). This feature makes them promising for application as ionizing radiation detectors for spectroscopic instruments.

**Figure 4:**
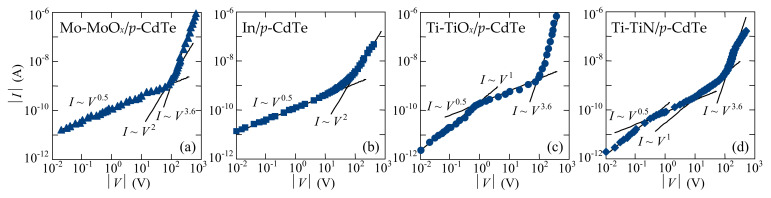
Reverse *I-V* characteristics of the (**a**) Mo-MoO*_x_*/*p*-CdTe/MoO*_x_*-Mo, (**b**) In/*p*-CdTe/MoO*_x_*-Mo, (**c**) Ti-TiO*_x_*/*p*-CdTe/MoO*_x_*-Mo, and (**d**) Ti-TiN/*p*-CdTe/MoO*_x_*-Mo Schottky-diode detectors in double logarithmic coordinates (symbols). Approximations of the square root (*I~V*^0.5^), linear (*I~V*), square-law (*I~V*^2^), and power-law (*I~V*^3.6^) voltage dependences of the current are shown by solid lines.

For evaluation of the mechanisms of charge transport in the fabricated Mo-MoO*_x_*/*p*-CdTe/MoO*_x_*-Mo, Ti-TiO*_x_*/*p*-CdTe/MoO*_x_*-Mo, Ti-TiN/*p*-CdTe/MoO*_x_*-Mo, and In/*p*-CdTe/MoO*_x_*-Mo heterostructures, the reverse *I-V* characteristics plotted in double logarithmic coordinates, have been analyzed (Figure 4). The specific regions in the voltage dependence of the current with different slopes clearly indicate alternating mechanisms of charge current transport. The initial parts of the *I-V* characteristics of the Mo-MoO*_x_*/*p*-CdTe/MoO*_x_*-Mo and In/*p*-CdTe/MoO*_x_*-Mo Schottky-diode detectors in the voltage ranges of 0.01 V < |*V*| < 100 V and 0.01 V < |*V*| < 60 V, respectively (Figure 4a,b), follow a square root dependence (*I ~ V*^0.5^) that evidences the generation nature of charge carrier transport in the space-charge region (SCR), described by the Sah–Noyce–Shockley theory [41,42].

**Figure 5:**
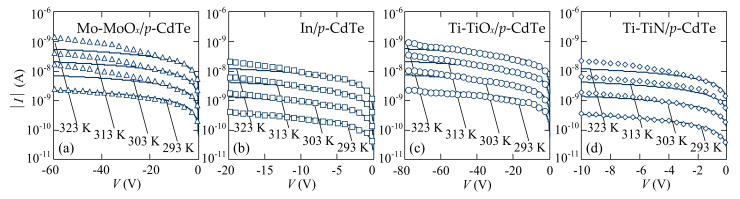
Comparison of the experimental data (symbols) and results calculated according to the Sah–Noyce–Shockley theory (solid lines) of the reverse *I-V* characteristics of the (**a**) Mo-MoO*_x_*/*p*-CdTe/MoO*_x_*-Mo, (**b**) In/*p*-CdTe/MoO*_x_*-Mo, (**c**) Ti-TiO*_x_*/*p*-CdTe/MoO*_x_*-Mo, and (**d**) Ti-TiN/*p*-CdTe/MoO*_x_*-Mo Schottky-diode detectors at different temperatures.

An additional confirmation of this assumption is provided by the comparison of the experimental *I-V* characteristics with the calculation results obtained in the framework of the Sah–Noyce–Shockley theory of generation–recombination of carriers, adapted to a metal-semiconductor (Schottky) contact (Figure 5). According to the theory [41], the generation current *I*_g_ can be found by integration of the generation rate *U*(*x*) throughout the entire SCR as shown in [17,21,22,23,24,25,42,43,44,45,46]:

(1) $$I_{g}=A\;q\int_{0}^{W}\frac{n\left(x,V\right)p\left(x,V\right)-n_{i}^{2}}{\tau_{p0}\left[n\left(x,V\right)+n_{1}\right]+\tau_{n0}\left[p\left(x,V\right)+p_{1}\right]}dx$$

where *A* is the diode area, *q* is the electron charge, *W* is the width of the SCR, *n*_i_ = (*N*_c_*N*_v_)^1/2^exp(−*E*_g_/2*kT*) is the intrinsic carrier concentration, *N*_c_ = 2(*m*_n_*kT*/2π*ħ*^2^)^3/2^ and *N*_v_ = 2(*m*_p_*kT*/2π*ħ*^2^)^3/2^ are the effective state densities in the conduction and valence bands, respectively, then *m*_n_ and *m*_p_ are the effective masses of electrons and holes, respectively, *τ*_p0_ and *τ*_n0_ are the effective lifetimes of holes and electrons in the SCR, *x* is the coordinate where an electron–hole pair is generated, and *k* is the Boltzmann constant. The values *n*_1_ and *p*_1_ are equal to the equilibrium concentrations of electrons and holes, respectively, under the condition that the Fermi level in the semiconductor coincides with the generation–recombination level with the ionization energy *E*_t_ (calculated from the top of the valence band). That is, *n*_1_ = *N*_c_exp(−*E*_t_/*kT*) and *p*_1_ = *N*_v_exp[−(*E*_g_−*E*_t_)/*kT*]. The values *n(x,V)* and *p(x,V)* are the concentrations of carriers in the conduction and valence bands within the SCR, respectively:

(2) $$n\left(x,V\right)=N_{c}\exp\left[-\frac{E_{g}-\Delta \mu -\phi \left(x,V\right)-qV}{kT}\right]\;\ldots \;p\left(x,V\right)=N_{v}\exp\left[-\frac{\Delta \mu +\phi \left(x,V\right)}{kT}\right]$$

where Δ*μ* is the energy of the Fermi level of the semiconductor.

The potential energy *φ(x,V)* of an electron in the SCR is described by the equation:

(3) $$\phi \left(x,V\right)=\left(\phi_{0}-qV\right){\left(1-\frac{x}{W}\right)}^{2}$$

where *φ*_0_ is the barrier height from the CdTe semiconductor side. The width of the SCR is expressed as [47]:

(4) $$W=\sqrt{\frac{2\varepsilon \varepsilon_{0}\left(\phi_{0}-qV\right)}{q^{2}N}}$$

where *ε* is the relative dielectric permittivity of the semiconductor and *ε*_0_ is the dielectric constant of vacuum.

The calculation results at different temperatures are shown by the solid line in the *I-V* characteristics of the heterostructures (Figure 5). The ionization energy of the generation–recombination center *E*_t_ was chosen to be equal to 0.69–0.70 eV, i.e., when the energy level of the center was located near the middle of the semiconductor bandgap (*E*_g_ = 1.47 eV at room temperature), as it was described by the Shockley–Read–Hall statistics [17,47].

The width of the SCR (Equation (4)) was calculated at the concentration of uncompensated acceptors *N* = *N*_a_−*N*_d_ = 5 × 10^10^ cm^−3^ as it was estimated for CdTe single crystals produced by Acrorad [20,21,22,23,24,25,26]. The temperature dependences of the bandgap *E*_g_*(T)* = 1.608 –4.52 × 10^−4^ × *T* (eV), holes mobility *µ*_p_ = 4 × 10^5^ × *T*^−3/2^ (cm^2^/V⋅s), and resistivity of CdTe were taken into account [15,16,20]. To ensure the best coincidence of the experimental data with the calculation results, the effective lifetime of electrons and holes in the SCR were chosen to be equal to *τ* = (τ_n0_τ_p0_)^1/2^ = 2 × 10^−7^ s. The voltage range in the calculations for each detector was used in which square root voltage dependences of the current were performed (Figure 4).

Since the magnitude of the generation current *I*_g_ is proportional to the concentration of intrinsic charge carriers in the semiconductor, the generation nature of the reverse current in the corresponding voltage range is further evidenced by the slope of the dependence log(*I*_g_*/T*^3/2^) vs. 10^3^/*T*. The analysis of such temperature dependences of the reverse current of the fabricated Mo-MoO*_x_*/*p*-CdTe/MoO*_x_*-Mo, In/*p*-CdTe/MoO*_x_*-Mo, Ti-TiO*_x_*/*p*-CdTe/MoO*_x_*-Mo, and Ti-TiN/*p*-CdTe/MoO*_x_*-Mo heterostructures at *V* = -2 V has shown that the slope equals 0.8 eV that corresponds to the half of the CdTe bandgap at *T* = 0 K [23,25]. Therefore, a very good agreement between the measurement and calculation results is observed, confirming that the model of the generation–recombination processes in the SCR adequately describes not only the voltage dependences of the current, but also the temperature-induced variations in the *I-V* characteristics of the CdTe-based detectors with different contact materials [21,22,23,24,25].

For the Ti-TiO*_x_*/*p*-CdTe/MoO*_x_*-Mo and Ti-TiN/*p*-CdTe/MoO*_x_*-Mo heterostructures, the generation nature of the reverse current is also observed in the voltage ranges of 0.6 V< |*V*| < 90 V and 0.3 V < |*V*| < 15 V, respectively (Figure 4c,d). A good agreement of the experimental and calculated results confirms this assumption (Figure 5c,d). However, the quite long initial linear regions (*I~V*^1^) of the reverse *I-V* characteristics precede the square root regions in the dependences *I*(*V*) (Figure 4c,d). This can be explained by the fact that the barrier height at the TiO*_x_*/*p*-CdTe and TiN/*p*-CdTe contacts (φ_0_ = 0.3 eV) is lower than that at the MoO*_x_*/*p*-CdTe and In/*p*-CdTe ones (φ_0_ = 0.6 eV). According to Equation (4), the depleted regions in the TiO*_x_*/*p*-CdTe and TiN/*p*-CdTe diode structures are thinner than those in the MoO*_x_*/*p*-CdTe and In/*p*-CdTe ones, and thus their resistances are lower. This circumstance explains the fact that the reverse currents flowed through the MoO*_x_*/*p*-CdTe and In/*p*-CdTe diode structures at low bias voltages are controlled by the reverse-biased Schottky contacts, whereas the initial linear regions of the reverse *I-V* characteristics of the TiO*_x_*/*p*-CdTe and TiN/*p*-CdTe diode structures are attributed to the fact that the resistance of the neutral (bulk) part of the CdTe crystal is comparable or even higher than the resistances of the depletion regions at low reverse bias. Therefore, the CdTe crystal bulk (neutral part) controls the reverse current in the initial regions of the *I-V* characteristics of the Ti-TiO*_x_*/*p*-CdTe/MoO*_x_*-Mo and Ti-TiN/*p*-CdTe/MoO_x_-Mo Schottky-diode detectors [21,22,23,24,25].

In view of the perspectives of employing the developed X/γ-ray detectors for spectroscopy, it is important that the *I-V* characteristic of the Ti-TiN/*p*-CdTe/MoO*_x_*-Mo Schottky-diode detector is linear in the voltage range of 15 V < |*V*| < 110 V (Figure 4d). Substitution *V* = −10 V into Equation (4) shows that the width of the SCR equals ~0.48 mm, i.e., at |*V*| > 10 V the depleted region occupies the entire thickness of the semiconductor crystal (*d* = 0.5 mm). In this case, the CdTe crystal behaves like a sample with a resistivity (*R* = (4–5) × 10^10^ Ω) higher than that of the bulk (neutral) part of the semiconductor (*R* = (6–7) × 10^9^ Ω). It is this circumstance, i.e., such current mechanism that leads to a linear voltage dependence of the current, which is observed as *I~V*^1^ at 15 V < |*V*| < 110 V (Figure 4d). As shown below (Section 5), this voltages range is the most optimal bias for using Ti-TiN/*p*-CdTe/MoO*_x_*-Mo detectors for spectroscopic measurements.

#### 3.2.2. Features of the Heterostructures with MoO*_x_* Ohmic Contacts at Higher Bias

However, a further increase in reverse bias (|*V*| > 100 V), applied to the Schottky-diode detectors with MoO*_x_* ohmic contacts, changes the current transport mechanism. The *I-V* characteristics of the Mo-MoO*_x_*/*p*-CdTe/MoO*_x_*-Mo and In/*p*-CdTe/MoO*_x_*-Mo heterostructures become proportional to the squared voltage (*I*~*V*^2^) (Figure 4a,b), i.e., the Mott–Gurney law for the space-charge-limited current (SCLC) is fulfilled. This is typical for semi-insulating materials [48,49]. Thus, the SCLC is expressed by:

(5) $$I_{\mathrm{SCLC}}=\theta \cdot \frac{9}{8}\frac{\varepsilon \varepsilon_{0}\mu }{d^{3}}V^{2}=\frac{N_{v}}{N_{t}}\exp\left(-\frac{E_{t}}{kT}\right)\frac{9}{8}\frac{\varepsilon \varepsilon_{0}\mu }{d^{3}}V^{2}$$

where *μ* is the mobility of charge carriers, *d* is the distance between the electrodes, i.e., the semiconductor crystal thickness. The factor *θ* takes into account the presence of accident impurities and point defects (traps), creating deep levels in the bandgap of a semiconductor. Such deep-level traps are inherent in CdTe because some number of uncontrolled impurities and corresponding intrinsic defects are always present in the semiconductor [3,4,5,6,7].

**Figure 6:**
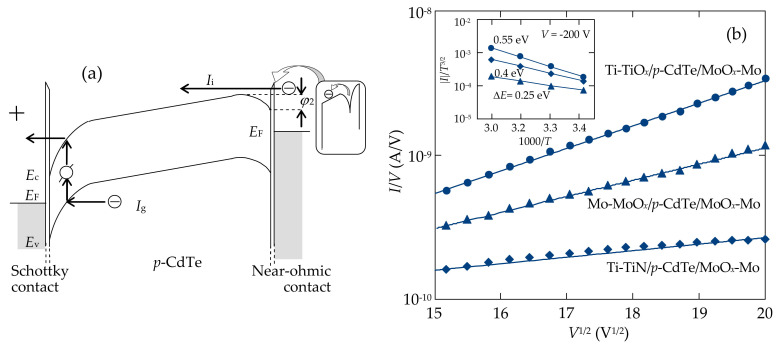
(**a**) Illustration of the Poole–Frenkel emission from the near-ohmic contact (CdTe/MoO*_x_*) showing the injection *I*_i_ and generation *I*_g_ currents (arrows) in the energy diagram of the reverse-biased CdTe-based heterostructure (the Schottky contact is biased positively with respect to the near-ohmic one). (**b**) Comparison of the results calculated according to the Poole–Frenkel emission model by Equation (6) (solid lines) with the measured *I-V* characteristics of the reverse-biased Mo-MoO*_x_*/*p*-CdTe/MoO*_x_*-Mo, Ti-TiO*_x_*/*p*-CdTe/MoO*_x_*-Mo, and Ti-TiN/*p*-CdTe/MoO*_x_*-Mo Schottky-diode detectors (symbols). The inset depicts the temperature dependence of the reverse currents in the heterostructures at *V* = −200 V.

Discussing the nature of excess concentration of charge carriers (necessary for space-charge-limited transport) in the Schottky-diode detectors under study, it is likely to explain the observed rapid increase in the current by the imperfection of the CdTe/MoO*_x_* ohmic contact. Then, if even a small downward band bending φ_2_ exists (Figure 6a), the CdTe/MoO*_x_* contact becomes forward-biased. Consequently, high injection of electrons from the ohmic contact takes place with a concentration much higher than the equilibrium concentration of carriers in the conduction band. In this case, the resistance modulation of the bulk (neutral) part of the CdTe crystal occurs [17,43]. This process is enhanced with increasing temperature. An agreement between the calculation results of the total reverse current *I = I*_g_ *+ I*_SCLC_ and experimental data was achieved for the trap level with the ionization energy *E*_t_ = 0.6 ± 0.02 eV.

The power-law voltage dependence of the reverse currents (*I ~ V*^3.6^) was observed at higher bias voltages: |*V*| > 125 V for the Mo-MoO*_x_*/*p*-CdTe/MoO*_x_*-Mo (Figure 4a) and Ti-TiN/*p*-CdTe/MoO*_x_*-Mo (Figure 4d) Schottky-diode detectors, and |*V*| > 90 for the Ti-TiO*_x_*/*p*-CdTe/MoO*_x_*-Mo sample (Figure 4c). This can be explained in the framework of the SCLC theory as the trap-filling limit [47,48,49]. According to the standard SCLC theory, the potential barrier height *E*_t_ of the trap in Equation (5) is unchanged for any electric field value applied to the diode structure. However, the activation energies of the reverse currents in the Mo-MoO*_x_*/*p*-CdTe/MoO*_x_*-Mo, Ti-TiN/*p*-CdTe/MoO*_x_*-Mo, and Ti-TiO*_x_*/*p*-CdTe/MoO*_x_*-Mo Schottky-diode detectors are lower (0.25–0.55 eV) at higher voltages (Figure 6b, inset). Therefore, at high electric fields, the barrier height *E*_t_ of the trap decreases, leading to the Poole–Frenkel electron emission from the oxide layer [50,51]. Such charge transport mechanism often explains the features of the electrical characteristics of metal-insulator-semiconductor structures [47,52]. The appearance of an oxide layer can occur after chemical polishing etching of the CdTe crystals in a K_2_Cr_2_O_7_ + HNO_3_ + H_2_O solution and further formation of thin Cd- or Te-rich surface layers [53,54].

According to the Poole–Frenkel model, the conductivity of the insulating film in the metal–insulator–semiconductor structure is due to thermal excitation of electrons from trapping centers located in the bandgap of the insulator (Figure 6a). Therefore, the temperature dependence of the conductivity is described by the factor exp(−*E*_t_*/kT*), where *E*_t_ is the trapping level energy (ionization energy of the trap at the absence of electric field). The lowering of the potential barrier height for electrons at the trapping center in high electric field is taken into account by an additional exponential factor exp(−(*q*^3^*V*/*πεε_0_d*_i_)^1/2^*/kT*), where *d*_i_ is the thickness of insulator. Therefore, the current can be expressed as [51,52]:

(6) $$I_{\mathrm{PF}}=Aq\mu_{n}\frac{V}{d_{i}}N_{c}\exp\left(-\frac{E_{t}}{kT}\right)\exp\left(-\frac{q\sqrt{qV/\pi \varepsilon \varepsilon_{0}d_{i}}}{kT}\right)$$

The square root voltage dependence in the exponent of the last factor is a feature of Poole–Frenkel emission [52]. As seen, the experimental data on the voltage dependence of the current presented in the corresponding coordinates are linear (Figure 6b).

This confirms the assumption about the nature of excess charge carriers. Thus, the Poole-Frenkel emission of electrons from the CdTe/MoO*_x_* near-ohmic contact to the semiconductor explains the increased electrical conductivity and the region with the dependence *I ~ V*^3.6^ in the *I-V* characteristics of the reverse-biased Mo-MoO*_x_*/*p*-CdTe/MoO*_x_*-Mo, Ti-TiO*_x_*/*p*-CdTe/MoO*_x_*-Mo, and Ti-TiN/*p*-CdTe/MoO*_x_*-Mo heterostructures at higher applied bias voltages (Figure 4a,c,d).

## 4. Capabilities of CdTe-Based X/γ-ray Detectors with an In Contact Treated by Laser Pulse Radiation

### 4.1. Laser-Assisted Formation of In/CdTe/Au Diode Structures

#### 4.1.1. Techniques of CdTe Crystal Surface Processing and Electrical Contact Formation

Prior to the formation of electrical contacts and electrode deposition, detector-grade (111) oriented CdTe single-crystal wafers were subjected to preliminary chemical surface processing for cleaning and removing of a disordered surface layer which was generally formed after mechanical polishing and storage of semiconductor crystals [53,54]. The CdTe samples were cleaned in acetone and methanol, then etched in a polishing 3–5% bromine–methanol solution, and finally were thoroughly rinsed with pure methanol. The polished and cleaned samples were dried in an argon flow before applying the next technological procedures of electrical contact formation and laser-induced doping.

**Figure 7:**
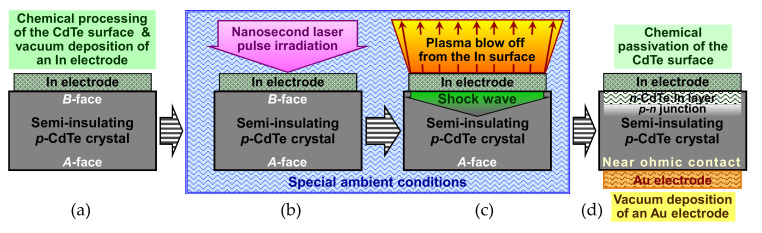
Schematic illustration of the fabrication procedures of In/CdTe/Au diode detectors using the laser irradiation technique: (**a**) chemical surface processing of the crystal and thermal vacuum deposition of an In electrode (dopant film) on the CdTe(111)*B* surface; (**b**) irradiation of the In/CdTe structure with nanosecond laser pulses; (**c**) laser-induced shock wave solid-phase doping of the *p*-CdTe surface region by In atoms (donors) and formation of a *p-n* junction; (**d**) chemical passivation of the crystal surfaces and thermal vacuum deposition of an Au electrode on the CdTe(111)*A* surface.

The techniques of the fabrication of the In/CdTe/Au diodes with a *p-n* junction are shown in Figure 7. After the preliminary surface processing, the CdTe crystals were subjected to low temperature (~70–90 °C) annealing in a vacuum chamber at low pressure (<0.6 mPa) for 1.5–2 h prior to before the metal contact deposition to remove a thin (~0.03 nm) Te film which was generally formed on the semiconductor surface during etching in bromine-containing solutions and then oxidized in air [53,54]. Then, an In film was evaporated on the CdTe(111)*B* crystal surface without heating of the samples (Figure 7a). The whole surface area of the CdTe crystal pre-coated with an In film was entirely and uniformly irradiated with nanosecond laser pulses at room temperature (Figure 7b). It was shown that distilled water environment was the optimal ambient condition for such treatment [55]. A KrF excimer laser, emitting single pulses with a wavelength of 248 nm and duration of 20 ns, was a pulsed radiation source. The incident laser pulse energy density was varied in a wide range both below and above the CdTe melting threshold [28]. A glass diffuser, homogenizer, and lens were used to provide uniform and controlled laser irradiation of the In/CdTe samples. The time and energy parameters of laser pulses were monitored during irradiation. The deposited In film was relatively thick (0.3–0.5 µm) and it was not completely evaporated under laser irradiation, thus the film served both as an *n*-type dopant source and electrical contact (electrode) after laser-induced doping (Figure 7c,d).

The second electrode was formed on the opposite side of the samples, i.e., on the CdTe(111)*A* surface by vacuum thermal evaporation of an Au film (0.3–0.5 µm) (Figure 7d). Both the In and Au electrodes were formed as 4 × 4 mm^2^ squares and centered on the Te- and Cd-terminated surfaces (5 × 5 mm^2^) of the sample, respectively. The deposition velocity and thickness of the electrodes (In and Au films) were controlled by the voltage applied to the evaporating metal source and monitored with an XTC thin film deposition controller. After laser irradiation of the In/CdTe structure from the In-coated side and prior to the Au electrode deposition, the sample was subjected to chemical passivation in an aqueous H_2_O_2_ solution, and then was rinsed with methanol (Figure 7d). Such procedure was employed to minimize lateral leakage currents, create the appropriate surface states at the CdTe(111)*A* side, and stabilize electrical characteristics of the In/CdTe/Au diode structure.

#### 4.1.2. Mechanisms of Laser Action on the In/CdTe Structure and *p-n* Junction Formation

The use of a relatively thick In electrode (0.3–0.5 µm), which also served as an dopant film, made it possible to ensure laser-induced doping without heating the underlying bulk In region and CdTe crystal that avoided a heat-induced deterioration of the structure and properties of the semiconductor. Despite the fact that absorption of laser radiation occurred in a thin (tens of nanometers) In surface layer, the thin semiconductor region under the In/CdTe interface was heavily doped with In atoms [55,56]. It was supposed that In/CdTe/Au diode structures with an In doped CdTe nano-layer and built-in *p-n* junction were obtained as a result of laser-stimulated modification of the defect structure and solid-phase doping in the deep-seated CdTe region.

Figure 7c illustrates the laser pulse action on the In/CdTe structure that was accompanied with superfast heating, melting, and evaporation of a thin (in order of the radiation absorption depth) In surface layer. The temperature of the laser-induced plasma (evaporated In and overheated environment medium, i.e., water) could reach 1000 °C and higher at laser pulse energy densities used in the experiment (80–130 mJ/cm^2^). Rapid expansion of the laser-heated In surface region and plasma recoil momentum resulted in generation of high-amplitude stress waves which were transformed to a high pressure shock wave [57]. Laser-induced stress and shock waves propagated through the In film and entered the CdTe crystal involving In dopant atoms [55,56]. Laser-stimulated penetration of high-concentration In dopants into the thin semiconductor region near the In-CdTe interface was due to essential elastic stress gradients, generation of stress and shock waves, and superfast diffusion of In atoms due to barodiffusion [56]. Despite high temperatures of the In electrode surface during laser irradiation, underlying deeper layers remained almost unheated because the deposited In film was much thicker than the laser-heated In surface region; therefore, the temperature in the bulk of In and particularly in CdTe did not significantly increased. This provided optimal conditions for solid-phase doping of a thin CdTe layer owing to rapid mass transport of the In impurity and transformation of the semiconductor point defect structure near the In/CdTe interface as a result of shock wave action and barodiffusion [56].

The fabricated In/CdTe/Au diode detectors were considered as complex metal-semiconductor multi-layered structures consisting of the following layers: In electrode, In/*n*-CdTe ohmic contact, low-resistivity highly doped *n*-CdTe:In layer, abrupt *p-n* junction, bulk part of semi-insulating *p*-CdTe, Au/*p*-CdTe near-ohmic contact and Au electrode (Figure 7d). Laser-induced doping of semi-insulating Cl-compensated *p*-like CdTe crystals was due to the modification of the point-defect system of the semiconductor [28,55]. Detector-grade high-resistivity CdTe:Cl semiconductor contains a large number of intrinsic and impurity point defects, in particular, V_Cd_ and Cl_i_, Cl_Te_ and other substitutional impurities in Te sites [3,4,5,6,7]. The point defects are generally aggregated as complexes (V_Cd_–Cl_Te_), (V_Cd_–2Cl_Te_) or (V_Cd_–Cl_i_) [6,7]. In detector-grade CdTe:Cl or CdTe:In crystals, complex acceptor defects called A-centers ((V_Cd_–Cl_Te_) and (V_Cd_–In_Cd_)) are typically formed that results in *p*-type conductivity of the semiconductors and, moreover, spontaneous formation of compensating acceptors (V_Cd_–In_Cd_) is the general problem in *n*-type doping of CdTe with an In impurity [6,58].

The main advantage of the modification of the CdTe structure and properties during laser irradiation of the crystals pre-coated with a relatively thick In film, was the action of an induced shock wave that could be considered as a stream of phonons scattered by point and extended defects of the crystalline structure. Such action resulted in the dissociation of defect complexes, barodiffusion of impurities and dopants, desorption, and segregation of interstitial atoms, etc. [28,55,56,57]. In the case of nanosecond laser irradiation of the In/CdTe structures, In dopant atoms, implicated by laser-induced stress and shock waves, penetrated into the CdTe region near the metal-semiconductor interface [28,55]. Laser stimulated processes of barodiffusion and migration of In atoms at V_Cd_ and then super-fast “freezing” of a large number of donor point defects (In_Cd_, Cd_i_, and Cl_Cd_) without formation of compensating acceptor complexes (V_Cd_–X), in particular A-centers like (V_Cd_–Cl_Te_) and (V_Cd_–In_Cd_), ensured solid-phase high In doping of a thin CdTe layer near the In/CdTe interface and formation of an abrupt *p-n* junction [28,55,56].

### 4.2. Electrical Characteristics of In/CdTe/Au Diode Structures Fabricated by Laser Irradiation

**Figure 8:**
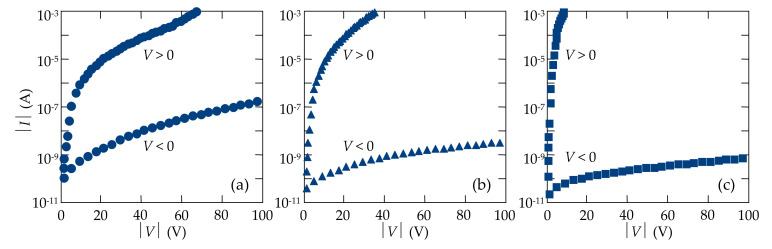
*I-V* characteristics of the In/CdTe/Au diode structures in semi-logarithmic coordinates (**a**) before and (**b**,**c**) after irradiation from the In electrode side by nanosecond pulses of a KrF excimer laser with energy densities of (**b**) 90 J/cm^2^ and (**c**) 120 J/cm^2^.

The In/CdTe/Au diode structures with a *p-n* junction, formed by laser irradiation of the In electrode, were examined by electrical measurements and then samples with low reverse dark currents were selected for testing them as X/γ-ray radiation detectors. Reverse current flowed when the In contact (near the *p-n* junction) was biased positively with respect to the Au contact (quasi-ohmic). Figure 8 shows the typical *I-V* characteristics of the In/CdTe/Au samples fabricated without laser irradiation of the In electrode (a) and with irradiation by nanosecond pulses of a KrF excimer laser with energy densities of ~90 J/cm^2^ (b) and ~110 J/cm^2^ (c). The *I-V* characteristics of the In/CdTe/Au diodes, measured in dark conditions at room temperature, showed excellent rectifying properties, especially taking into account the fact that CdTe crystals used for detector fabrication were semi-insulating (Figure 8).

In the case of unirradiated In/CdTe/Au samples with just deposited In and Au electrodes (Figure 8a), the rectification was due to a high Schottky barrier at the In/CdTe interface that was typical for an In electrical contact and semi-insulating *p*-like CdTe and this was widely used for fabrication of Schottky diode detectors [5,6,7,8,9,10,14]. Moreover, the rectification significantly increased after laser irradiation of the In/CdTe structure (Figure 8b,c). As discussed in the section above, it resulted from laser-induced doping of the thin CdTe region under the In/CdTe interface with an In donor dopant and formation of a shallow and abrupt *p-n* junction according to the doping mechanisms investigated earlier [28,55,56]. As seen, laser irradiation of the In/CdTe structure from the In electrode side remarkably shifted the *I-V* characteristic forward branch toward lower voltages (forward current increased) and reduced reverse current compared with the unirradiated In/CdTe/Au sample (Figure 8). From a practical point of view, it is important to note that the reverse current of the In/CdTe/Au *p-n* junction diodes reduced by more than 250 times due to laser treatment of the In electrode (Figure 8c).

**Figure 9:**
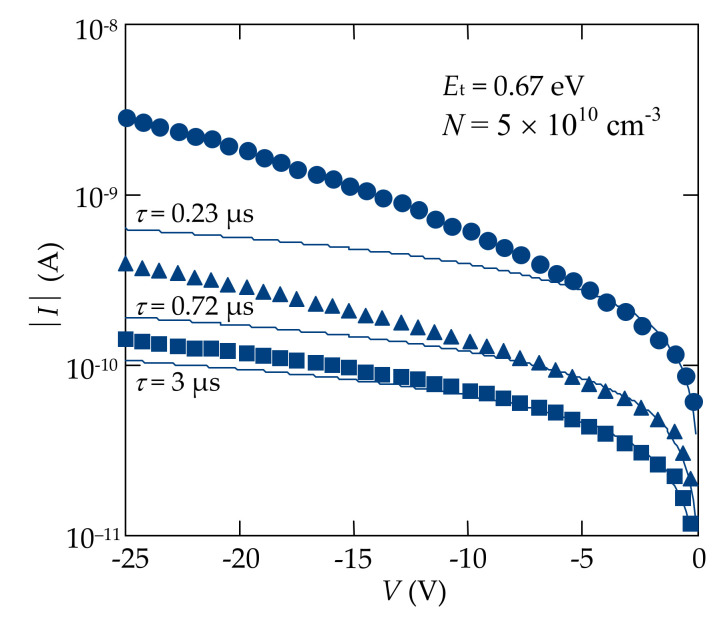
Comparison of the experimental (symbols) and calculated according to the Sah–Noyce–Shockley theory (solid lines) results of the reverse dark *I-V* characteristics of the In/CdTe/Au diode structures before (circles) and after (triangles and squares) irradiation from the In electrode side by nanosecond pulses of a KrF excimer laser with energy densities of 90 J/cm^2^ (triangles) and 120 J/cm^2^ (squares).

Figure 9 presents a comparison of the calculation results using Equations (1)–(4) (solid lines) and experimental data (symbols). It should be emphasized that the calculations were performed at the concentration of uncompensated acceptors *N = N*_a_–*N*_d_ = 5 × 10^10^ cm^−3^ as it corresponded to CdTe single crystals produced by Acrorad and the ionization energy of the generation–recombination center was accepted as *E*_t_ *=* 0.67 eV [20,21,22,23,24,25,26]. The computed results exhibited that the lifetimes *τ*_n0_ of electrons and holes *τ*_p0_ were such critical parameters determining the reverse current values in the *I-V* characteristics.

As mentioned in the section above, the laser-generated stress and shock waves penetrated into the semiconductor region near the In/CdTe interface, implicating In atoms and introducing them as a dopant into the crystal lattice, that decreased the concentration of vacancies in that region. In particular, Cd vacancies were partly filled by the nearest accidental impurities, mainly by In dopant atoms [28,55]. In atoms, substituting Cd atoms, acted as donors [6,58]. So, the impact of laser irradiation increased the effective lifetimes of charge carriers in the depleted region, reduced the generation rate, and thus decreased the reverse dark current of In/CdTe/Au *p-n* junction detectors.

As seen from Figure 9, the Sah–Noyce–Shockley theory describes well the reverse dark current in the unirradiated In/CdTe/Au diode only in the voltage range of 0 V < |*V*| < 5 V (circles). The sections of the plots, where the experimental results coincide with calculations, expand in In/CdTe/Au diode structures formed by laser irradiation. In particular, the generation current prevails in the In/CdTe/Au diode after laser treatment with energy density of 90 J/cm^2^ in the voltage range of 0 V < |*V*| < 10 V (triangles), whereas laser irradiation with energy density of 120 J/cm^2^ leads to an increase in the voltage range, corresponding to the generation current, up to ~ −15 V (squares) (Figure 9).

## 5. Spectroscopic Characteristics of CdTe-Based X/γ-ray Detectors

The spectroscopic performance, including the energy resolutions of the fabricated CdTe-based detectors, was examined using ^241^Am (59.5 keV), ^57^Co (122 keV), and ^137^Cs (662 keV) isotopes as X/γ-ray radiation sources as well as employing a portable spectrometer ANS-MNT004-GTK produced by ANSeeN, Inc. or standard laboratory equipment (a charge-sensitive preamplifier 5102 BS produced by Clear Pulse Co., Ltd. (Tokyo, Japan), coupled to a multichannel analyzer MCA7600 produced by Seiko EG&G Co., Ltd. (Tokyo, Japan), etc.) in the case of investigation of the In/CdTeAu *p-n* junction diode detectors. The spectroscopic measurements were carried out at room temperature. The electrodes (quasi-ohmic contact) deposited on the CdTe(111)*A* surface (Cd-terminated) were biased negatively for all the tested detectors.

The energy resolution of the developed detectors was determined by the fabrication techniques and materials selected for the electrical contact formation as well as was depended on the applied bias voltage. The voltage dependences of the energy resolution (FWHM) slightly differed for CdTe-based detectors created by the same techniques, but under different conditions. It can be explained by the decreasing of the misfit strains at the interface between the thin contact film and bulk crystal. In particular, for the Mo-MoO*_x_*/*p*-CdTe/MoO*_x_*-Mo, Ti-TiO*_x_*/*p*-CdTe/MoO*_x_*-Mo, Ti-TiN/*p*-CdTe/MoO*_x_*-Mo, and In/*p*-CdTe/MoO*_x_*-Mo structures the misfit strain was determined by well-known ratio [59] and equals to 14%, 29%, 34%, and 18%, respectively. Another reason for differences in the energy resolutions could be due to different surface states at the CdTe crystal surfaces when electrical contacts were deposited [21,22,23,24,25]. The optimal bias voltages were found for each detector with MoO*_x_* contacts at which the energy resolution and detection efficiency reached the best values.

In Table 1, the energy resolution values for the 59.5 keV and 662 keV peaks in the spectra of ^241^Am and ^137^Cs isotopes are respectively presented for the detectors with different contact materials at different combinations of applied bias voltages. For the CdTe-based structures with the same electrical contacts, the energy resolutions varied from the detector to detector by ~25%. At lower bias voltages, the energy resolution degraded due to insufficient field strength to collect photogenerated charge carriers and at higher voltages, deterioration in the energy resolution was due to excessive dark current in the detectors. Some of the reasons for such features of the detectors was the effect of the CdTe crystal defect structure and misfit strains in the transition layer at the electrical contact-bulk semiconductor interface. Here, the results for the best performance of the detectors with one MoO*_x_* contact and the second one made from different materials are presented.

**Figure 10:**
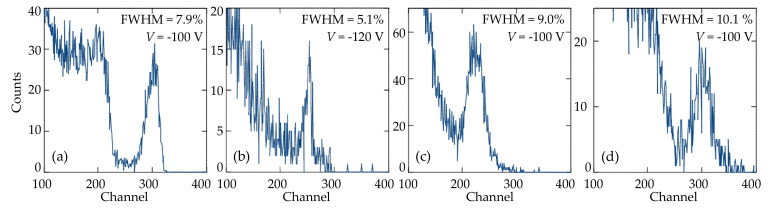
Room temperature spectra of a ^137^Cs (662 keV) isotope taken with the (**a**) Mo-MoO*_x_*/*p*-CdTe/MoO*x*-Mo, (**b**) In/*p*-CdTe/MoO*_x_*-Mo, (**c**) Ti-TiO*_x_*/*p*-CdTe/MoO*_x_*-Mo, and (**d**) Ti-TiN/*p*-CdTe/MoO*_x_*-Mo Schottky-diode detectors.

**Figure 11:**
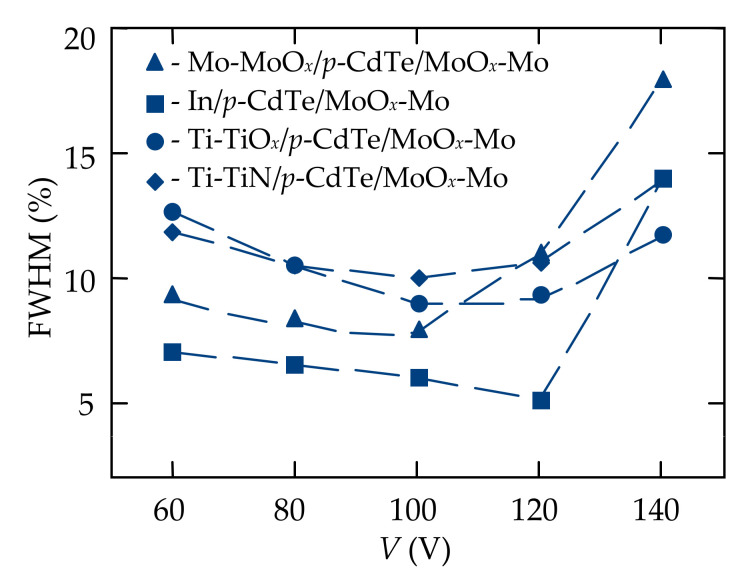
Effect of the bias voltage applied to the Mo-MoO*_x_*/*p*-CdTe/MoO*_x_*-Mo (triangles), In/*p*-CdTe/MoO*_x_*-Mo (squares), Ti-TiO*_x_*/*p*-CdTe/MoO*_x_*-Mo (circles), and Ti-TiN/*p*-CdTe/MoO*_x_*-Mo (diamonds) Schottky-diode detectors on the energy resolution (FWHM) of the 662 keV photopeak in the room temperature ^137^Cs isotope spectra.

The energy resolution and efficiency of the fabricated CdTe-based detectors depended on the applied bias voltage (Figure 10 and Figure 11). Figure 10 shows the spectra of a ^137^Cs (662 keV) isotope with the best values of FWHM, measured by the fabricated heterostructures with different electrical contacts at the optimal bias voltages. As seen, the highest resolution (the lowest value of FWHM = 5.1%) was obtained for the In/*p*-CdTe/MoO*_x_*-Mo Schottky-diode detector at *V* = −120 V (Figure 10b). However, the energy resolution deteriorated (FWHM rose) with increasing bias voltage that was due to a sharp increase in the dark (leakage) current of the diode (Figure 4b).

The techniques developed for the modification of the surface state of semi-insulating *p*-like CdTe crystals and electrical contact formation, using nanosecond pulse laser irradiation, resulted in the optimization of the detector fabrication technology that made it possible to obtain In/CdTe/Au diode-type X/γ-ray sensors with higher detection efficiency and energy resolution in comparison with the Mo-MoO*_x_*/*p*-CdTe/MoO*_x_*-Mo, In/*p*-CdTe/MoO*_x_*-Mo, Ti-TiO*_x_*/*p*-CdTe/MoO*_x_*-Mo, and Ti-TiN/*p*-CdTe/MoO*_x_*-Mo Schottky-diode detectors, which we also elaborated, fabricated and investigated. However, CdTe-based detectors with molybdenum oxide ohmic contacts and titanium oxide, titanium nitride, and indium Schottky contacts have shown promise and it is possible to achieve better performance by modification and optimization of the technology procedures of CdTe crystal surface preparation and electrode deposition techniques using different contact materials [21,22,23,24,25,35,36,37,38,39,40].

**Figure 12:**
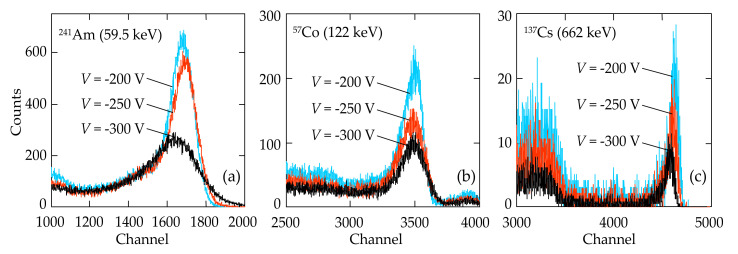
Room temperature spectra of (**a**) ^241^Am (59.5 keV), (**b**) ^57^Co (122 keV), and (**c**) ^137^Cs (662 keV) isotopes at different bias voltages (−200 V, −250 V, and −300 V) taken with the In/CdTe/Au *p-n* junction detector, fabricated by laser radiation of the In electrode.

Due to creating the appropriate conditions for photogenerated charge carrier collection, lowering the reverse dark (leakage) current in the In/CdTe/Au *p-n* junction diode detectors and thus increasing detecting ability and decreasing electrical noises in the detectors, the spectra of ^241^Am, ^57^Co, and ^137^Cs isotopes with quite high energy resolutions were obtained (Figure 12). There was also found a certain optimal bias voltage range for each In/CdTe/Au *p-n* junction detector that provided higher numbers of counts (better detection efficiency), lower FWHM values for the isotope spectrum photopeaks (higher energy resolution), and true peak channel positions (Figure 12).

Table 2 presents the data on the energy resolutions of the In/CdTe/Au *p-n* detector calculated for the emission spectra of three isotopes measured at different bias voltages (Figure 12). As seen, the best FWHM values were obtained in the spectra taken at *V* = −200 V. A quite symmetrical shape of the prominent lines of 59.5 keV, 122 keV, and 662 keV in the spectra of ^241^Am, ^122^Co and ^137^Cs isotopes, respectively, for all the applied bias voltages evidences that the full charge collection was complete even at the lowest bias voltage *V* = −200 V (Figure 12). The broad shoulder at the low-energy side from the 662 keV line (c) was attributed to Compton scattering of γ-rays [7,8,9,10].

An increase in bias voltage (*V* = −250 V and *V* = −300 V) resulted in deterioration of the spectra: the number of counts decreased (a–c), the FWHM values increased (a–c) and the peak channel positions for the 59.5 keV (a) and 662 keV (c) lines were shifted toward the lower-energy side (Figure 12). Such distortion in the isotope spectra with rising bias voltages was attributed to an increase in the total reverse current, i.e., photocurrent (charge packet) and leakage current (mainly due to lateral currents because no any guard ring was used in the experiments).

The features of the CdTe-based X/γ-ray detectors with different contact materials were studied in details earlier [18,19,20,21,22,23,24,25,26,27,28,29,30,31,32,33,34]. The capabilities of the developed detectors to operate for long time without deterioration of their functional parameters were shown and analyzed. However, further improvement of spectroscopic properties of CdTe-based detectors can be achieved by the optimal choice of contact materials, pre-treatment procedures of the semiconductor crystal surfaces, and corresponding techniques of electrode deposition.

## 6. Conclusions

Based on the developed efficient techniques of semiconductor crystal surface processing and electrode deposition, X/γ-ray detectors with different contact materials were fabricated using the same detector-grade *p*-like CdTe produced by Acrorad Co. The heterostructures with ohmic (MoO*_x_*) and Schottky-type (MoO*_x_*, TiO*_x_*, TiN, and In) contacts, created by DC reactive magnetron sputtering and vacuum thermal evaporation, were characterized by electrical and spectroscopic measurements. It was shown the possibility of the application of molybdenum oxide thin films, as an ohmic or Schottky contacts to semi-insulating *p*-CdTe crystals, depending on pre-treatment of their surfaces. The fabricated Mo-MoO*_x_*/*p*-CdTe/MoO*_x_*-Mo, In/*p*-CdTe/MoO*_x_*-Mo, Ti-TiO*_x_*/*p*-CdTe/MoO*_x_*-Mo, and Ti-TiN/*p*-CdTe/MoO*_x_*-Mo Schottky-diode detectors showed high rectification properties with quite low reverse dark currents and were sensitive to X/γ-ray radiation with moderate energy resolutions (5–20%@59.5 keV, 5–10%@662 keV). The comparative analysis of the *I-V* characteristics of the developed heterostructures in the framework of the well-known theoretical models allowed us to determine the dominant mechanisms of charge carrier transport and reasons limiting the efficiency and energy resolution of the heterostructure-based X/γ-ray detectors. The charge transport mechanisms, dominating in the heterostructures at certain bias voltage ranges, were attributed to the generation–recombination in the SCR at low bias voltages, SCLC at higher voltages, and the Poole–Frenkel emission at the highest applied biases. The last effect limited the use of the developed Schottky-diode heterostructures as X/γ-ray detectors at high bias voltages.

Higher energy resolutions (7–15%@59.5 keV, 4–6%@122 keV, and 1.6–3%@662 keV) were obtained by the developed In/CdTe/Au diode detectors with a *p-n* junction formed by laser-induced doping of a thin CdTe surface layer with In atoms (donors). This was realized by irradiation of the *p*-CdTe crystals pre-coated with an In dopant film with nanosecond laser pulses. The analysis of the *I-V* characteristics of the In/CdTe/Au structures evidenced that laser irradiation of the In electrode increased the bias voltage range corresponding to the charge transport mechanism of the carrier generation–recombination in the SCR.

The use of different contact materials and modification of the methods and techniques of surface processing of semiconductor crystals, contact formation, and electrode deposition expand the possibilities to achieve better performance of room temperature CdTe-based X/γ-ray detectors.

## Figures and Tables

**Table 1:** Energy resolutions of the CdTe-based detectors with different contact materials, measured at different bias voltages for two X/γ-ray radiation sources.

X/γ-ray Source	Anode/CdTe/Cathode	Bias Voltage (V)	FWHM (%)
^241^Am (59.5 keV)	Mo-MoO*_x_*/*p*-CdTe/MoO*_x_*-Mo	−80	6
	Ti-TiO*_x_*/*p*-CdTe/MoO*_x_*-Mo	−100	>20
	Ti-TiN/*p*-CdTe/MoO*_x_*-Mo	−80	11
	In/*p*-CdTe/MoO*_x_*-Mo	−100	>25
^137^Cs (662 keV)	Mo-MoO*_x_*/*p*-CdTe/MoO*_x_*-Mo	−100	7.9
	Ti-TiO*_x_*/*p*-CdTe/MoO*_x_*-Mo	−100	9
	Ti-TiN/*p*-CdTe/MoO_x_-Mo	−100	10.1
	In/*p*-CdTe/MoO*_x_*-Mo	−120	5.1

**Table 2:** Energy resolutions of the In/CdTe/Au *p-n* junction detector, fabricated by laser radiation of the In electrode, measured at different bias voltages for three X/γ-ray radiation sources.

**X-ray Source**	**Bias Voltage (V)**		
	−200	−250	−300
	**FWHM (%)**		
^241^Am (59.5 keV)	7.5	9.04	14.72
^57^Co (122 keV)	4.79	5.62	6.19
^137^Cs (662 keV)	1.6	2.1	2.7
